# Supplementary material for: Morphology Effect of Zinc Oxide Nanoparticles on the Gas Separation Performance of Polyurethane Mixed Matrix Membranes for CO2 Recovery from CH4, O2, and N2
Source: Membranes (Basel). 2022 May 31;12(6):577. doi: 10.3390/membranes12060577 (PMC9230613; doi:10.3390/membranes12060577)
Supplement: Supplementary file 1 [file membranes-12-00577-s001.zip › membranes-1735527-supplementary.pdf]

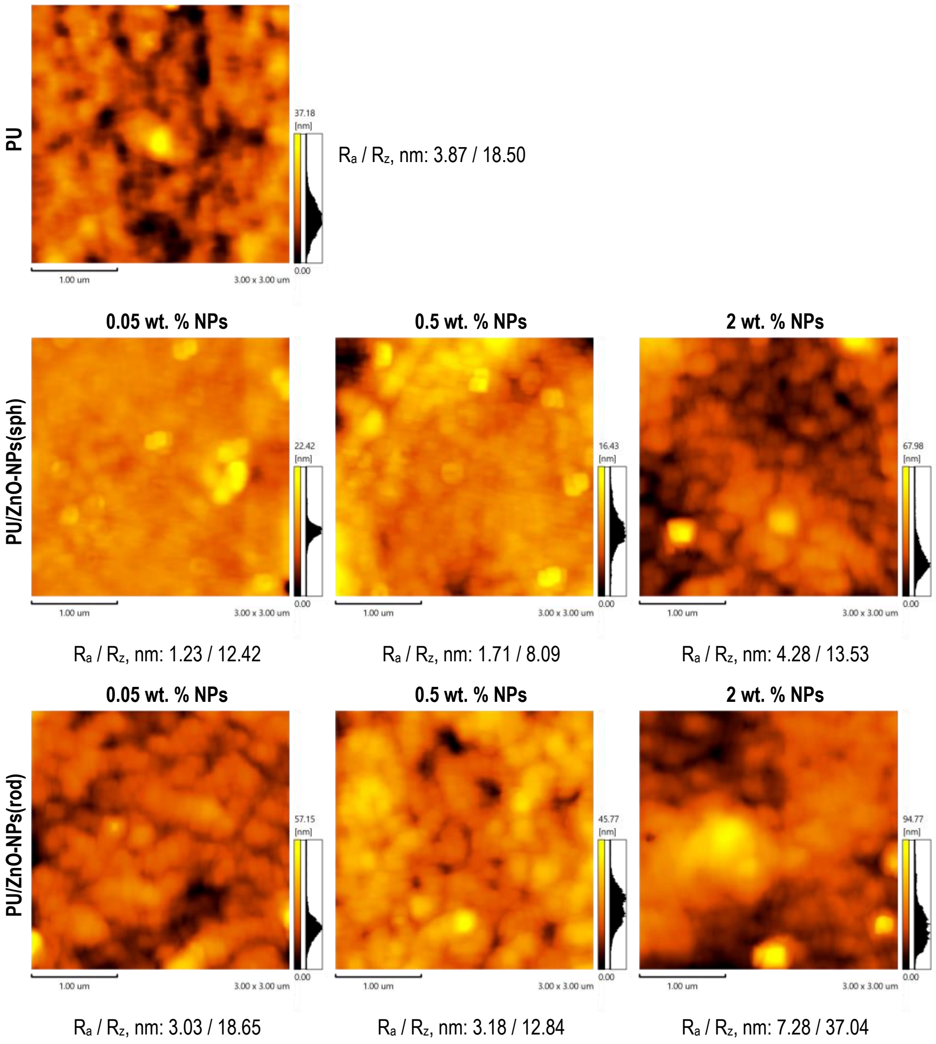

Figure S1. The AFM data of the reverse side of the membranes based on the various PU solutions with ZnO-NPs.
